# Antigen Coverage Presented by MHC Class I Has a Negative Correlation with SARS-CoV-2-Induced Mortality

**DOI:** 10.3390/vaccines10111917

**Published:** 2022-11-13

**Authors:** Ji Soo Park, Kwoneel Kim

**Affiliations:** 1 Department of Biology, Kyung Hee University, Seoul 02447, Korea; 2 Department of Biomedical and Pharmaceutical Sciences, Kyung Hee University, Seoul 02447, Korea

**Keywords:** COVID-19-induced mortality, MHC class I, SARS-CoV-2 antigen coverage, HLA-B, SARS-CoV-2 spike domain

## Abstract

The COVID-19 pandemic has caused a health crisis worldwide; therefore, it is necessary to understand the factors related to its prognosis. In this study, we hypothesized that SARS-CoV-2-derived antigens presented by MHC class I may correlate with mortality in COVID-19 because they induce adaptive immune responses. Antigen coverage at the national level was inferred using country-specific HLA allele frequencies and relative predictions of binding antigens. We performed regression analysis between antigen coverage and the death rate due to COVID-19 across countries and found a negative correlation, although it was statistically significant only in HLA-B. This negative correlation was corroborated in multiple regression analysis with known risk factors, such as the prevalence of underlying disease. Furthermore, we analyzed antigen coverage in accordance with SARS-CoV-2 domains and identified a significant negative correlation when it was derived from the spike domain, which is reported to be favorable for COVID-19 prognosis. Taken together, the results indicate that the antigen coverage of SARS-CoV-2 specifically presented by HLA-B may act as a favorable factor when explaining COVID-19-induced mortality

## 1. Introduction

Human infection by SARS-CoV-2 started at the beginning of December 2019, and in January 2020, the World Health Organization (WHO) declared an international public health emergency. As of March 2022, the cumulative number of confirmed patients exceeded 400 million, with deaths exceeding 6 million. Currently, international shutdowns and the economic shockwaves caused by the COVID-19 pandemic is the most urgent problem to solved. With this background, genetic effects on COVID-19 severity have been studied on the mechanism of the immune response [1]. Innate immunity and adaptive immune responses are invoked when an unrecognized virus enters the body. The ability to induce adaptive immune responses largely depends on antigen presentation through human leukocyte antigen (HLA) class I and II molecules [2]. HLA molecules are known to be involved in the antigen presentation process of the acquired immune system and is a representative immune-related factor. HLA genes encode a cell membrane glycoprotein molecule expressed on the surface of a human nucleated cell that binds to an antigen inside the cell and then presents it to the T lymphocytes, causing an adaptive immune response to the antigen [3]. In the context of the COVID-19 pandemic, susceptibility to severe disease may be related to the presentation of SARS-CoV-2-derived antigens by HLA molecules [4]. For example, infection by MERS-CoV belonging to the same coronavirus family elicits more severe symptoms in a person who carried the HLA-B 46:01 genotype [5]. Studies on antigens presented by relative HLAs could reveal high-risk groups and consider candidate peptides for vaccination strategies [6]. Therefore, this study analyzed the correlation between SARS-CoV-2-induced death and HLA alleles based on their frequency in human populations.

We sought to analyze the antigen coverage of SARS-CoV-2 presented by human MHC class I, which is composed of HLA-A, -B, and -C and exhibits the highest degree of polymorphisms among human genes, with many types of alleles [7]. Overall, the binding affinity of antigens varies considerably according to the type of HLA allele. Hence, specific HLA alleles have been reported to have a positive correlation with a favorable response to virus infection, with the potential to present more virus antigens [2]. This is because more presentation of virus antigen provokes a more effective immune response. Wide HLA polymorphism manifests as different allele frequencies across countries, and one study revealed that the difference in HLA alleles is associated with the mortality rate of COVID-19 [8]. Accordingly, we hypothesized that different HLAs by country cause differences in antigen presentation of SARS-CoV-2, resulting in different prognoses.

## 2. Materials and Methods

### 2.1. Epidemiological Data

The epidemiological data used in this analysis (number of total cases, total deaths, total cases per 1 million inhabitants, total deaths per 1 million inhabitants) were retrieved from the World Meter database (www.worldometers.info/coronavirus, accessed on 12 January 2021). This database collects data from official reports, directly from government communication channels and indirectly through local media sources when deemed reliable. In total, 41 countries with more than 1000 total infection cases and an infection rate of less than 10% of the population were evaluated. Two covariates, sex and age, were included in the regression analysis with risk factors, and they are represented as the male ratio and median value. Data on asthma, chronic kidney disease, liver disease, type 1 diabetes, and type 2 diabetes rates among risk factors selected by the Centers for Disease Control and Prevention are available in a public, open access repository (https://ghdx.healthdata.org/gbd-results-tool, accessed on 1 November 2021).

### 2.2. HLA Class I

Human leukocyte antigen (HLA) I alleles for A and B gene frequencies in the selected populations were retrieved from the Allele Frequency Net Database (http://www.allelefrequencies.net, accessed on 18–26 January 2021). Larger datasets that did not include minority ethnic groups and with at least 100 individuals were preferred. Datasets were graded at least silver or higher. Gold, silver or bronze (GSB) standards were a classification according to sample size, level of resolution at typing and frequency data. Countries with two or more datasets were combined with absolute consideration of the sample size.

### 2.3. T Cell Epitope Prediction for SARS-CoV-2

Full-length viral nucleotide sequences of SARS-CoV-2 (accession number NC_045512) were downloaded from NCBI GenBank. Binding affinity to HLA class I molecules was calculated for all 9- and 10-mer peptides from SARS-CoV-2 proteins using the Python environment v3.8 and the MHCflurry v2.0 [9] and NetMHCpan v4.1 tools [10]. Only peptides with binding affinity less than 500 nM were agreed to bind with HLA.

### 2.4. Antigen Coverage Calculation

Antigen coverage was calculated and used as a surrogate for the degree of antigen presentation at the country level. The number of SARS-CoV-2 binding antigens for each HLA allele was predicted using MHCflurry and NetMHCpan, after which national allele frequencies of HLAs were combined with the predicted antigen number to be presented by HLAs. The antigen coverage for HLA-A or HLA-B was obtained by the summation of antigen coverages for the relevant alleles. A similar approach was adopted for this antigen coverage calculation [11].

## 3. Results

### 3.1. Antigen Coverage of SARS-CoV-2 Correlated with the Death Rate of COVID-19

To study the correlation between the antigen coverage of SARS-CoV-2 and the prognosis of its infection at the population level, we obtained country-specific allele frequencies of HLA-A and HLA-B from a reliable public database (http://www.allelefrequencies.net) with a sufficient population (*n* ≥ 100). Candidate SARS-CoV-2 antigens were generated by sliding windows of 9 or 10 amino acids using the SARS-CoV-2 reference genome (NC_045512) because the size of the antigen presented by HLAs is known to be between 8 and 15 amino acids. We used MHCflurry [9] and NetMHCpan [10] algorithms to predict antigen presentation via HLA molecule binding. The antigens predicted as having strong HLA binding were identified to calculate antigen coverage across countries. We inferred the antigen coverage using country-specific HLA allele frequencies and relative predictions of antigen presentation, as the information for HLA and antigen presentation for each infected individual was limited (see detailed information in Materials and Methods). The number of deaths from COVID-19 was obtained from a public database (www.worldometers.info/coronavirus accessed on 16 January 2021) that collects the prevalence and mortality of COVID-19 worldwide.

**Figure 1:**
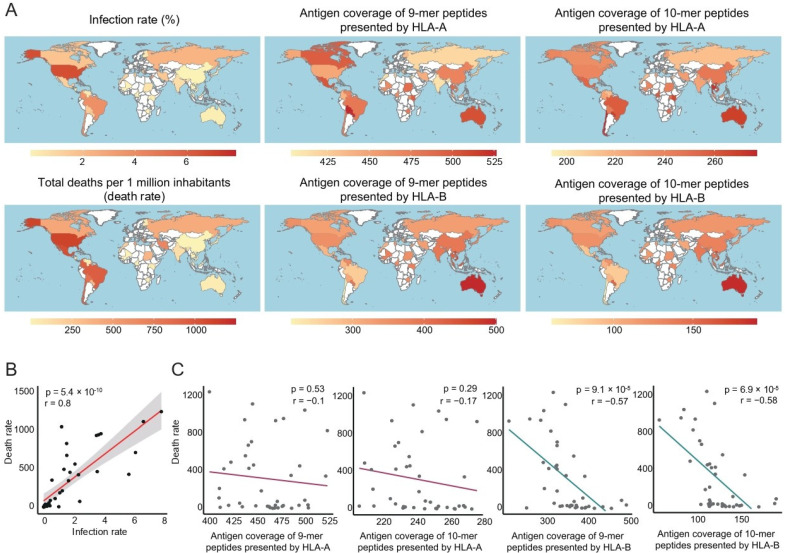
(**A**) Global heatmaps show antigen coverage of SARS-CoV-2, infection rate, and mortality of COVID-19 for each country. The antigen coverage in all figures is the result predicted by MHCflurry. (**B**) The results of linear regression of the COVID-19 infection rate and mortality for each country. The *X*-axis represents the infection rate, and the *Y*-axis represents the mortality rate. (**C**) The results of linear regression of antigen coverage and mortality. The *X*-axis represents antigen coverage, and the *Y*-axis represents mortality. The mortality rate was estimated as the number of total deaths per 1 million inhabitants. The red and blue lines indicate the correlation coefficient of mortality with the antigen coverage of HLA-A and HLA-B, respectively.

We first investigated the global distribution of antigen coverage of SARS-CoV-2 along with the infection and death rates (Figure 1A and Figure S1A). The SARS-CoV-2 infection rate showed a positive correlation with the COVID-19 death rate but had a negative correlation with antigen coverage. We confirmed the positive correlation between the infection rate and death rate by regression analysis (Figure 1B, r = 0.8, *p* < 0.05). We then analyzed the association between death rate and antigen coverage by the same regression analysis, and antigen coverage showed a negative correlation with the death rate regardless of its inference, even though some of the analyses did not show a statistical significance (Figure 1C and Figure S1B).

### 3.2. Antigen Coverage Correlated Negatively with COVID-19 Mortality, in Contrast to Other Risk Factors

**Figure 2:**
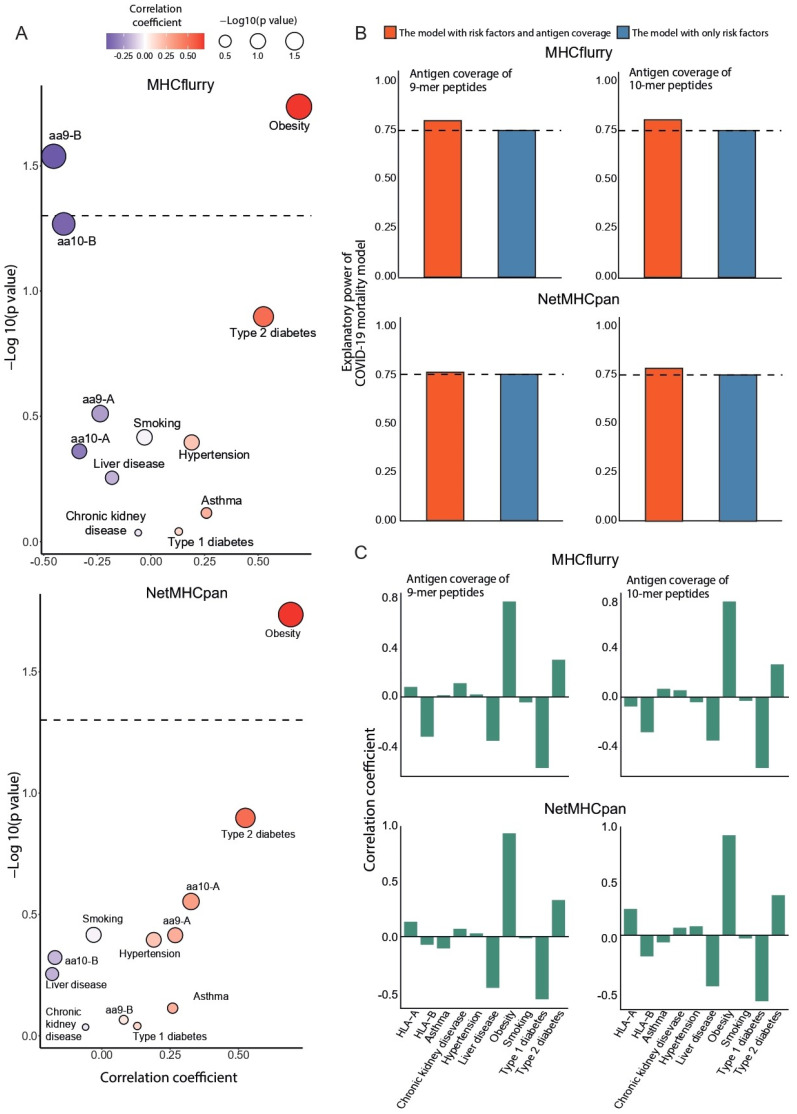
(**A**) Eight risk factors (asthma, chronic kidney disease, liver disease type 1 diabetes mellitus, type 2 diabetes mellitus, hypertension, obesity, smoking) and the antigen coverage of HLA-A and HLA-B were analyzed for their correlation with the death rate of COVID-19. The dot color and *X*-axis represent correlation coefficients, and the size and *Y*-axis represent *p* values. The dotted line indicates *p* value = 0.05. (**B**) The figure shows the r squared value of linear the regression model between mortality rate and risk factors. We compared the model constructed by risk factors (red bar) to the model extended by the antigen coverage of HLA-A and HLA-B (blue bar). We estimated antigen coverage by using 9-mer (left) and 10-mer peptides (right). The dotted line represents the r squared value of the regression model constructed using only risk factors. (**C**) The multivariate linear regression results for the mortality rates related to risk factors and antigen coverage, showing the coefficients of each risk factor and antigen coverage. We estimated antigen coverage by using 9-mer (left) and 10-mer peptides (right). The antigen coverage in Figure 2 was calculated based on the prediction of antigen presentation using MHCflurry and NetMHCpan.

The Centers for Disease Control and Prevention (CDC) have reported several risk factors for adverse outcomes in COVID-19. We collected the national prevalence rate for eight risk factors. The prevalence rates of asthma, chronic kidney disease, liver disease, type 1 diabetes mellitus, and type 2 diabetes mellitus are available in the open access repository (https://ghdx.healthdata.org/gbd-results-tool accessed on 1 November 2021); rates of hypertension [12], obesity, and smoking were obtained from public data (see detailed information in Section 2). Regression analyses of individual risk factors and antigen coverage were performed with the death rate of COVID-19 to compare their effects on COVID-19 prognosis. After adjustment for sex and age [13] as covariates, the rate of obesity showed a positive correlation with the death rate of COVID-19 at a statistically significant level. Conversely, antigen coverage presented by HLA-B correlated significantly negatively with mortality (Figure 2A). Additionally, we next constructed an integrative regression model encompassing risk factors and antigen coverage to explain the mortality of COVID-19. Interestingly, the explanatory power of the multivariate regression model generated by only risk factors increased when antigen coverage was added to the regression model (Figure 2B). Furthermore, the antigen coverage specifically presented by HLA-B showed a negative effect in the multivariate model, in agreement with its negative effect in the univariate regression model (Figure 2C). These results suggest that antigen coverage may act as a favorable factor for the prognosis of COVID-19.

### 3.3. Effect of Antigen Coverage on the Mortality of COVID-19 and RNA Domains

**Figure 3:**
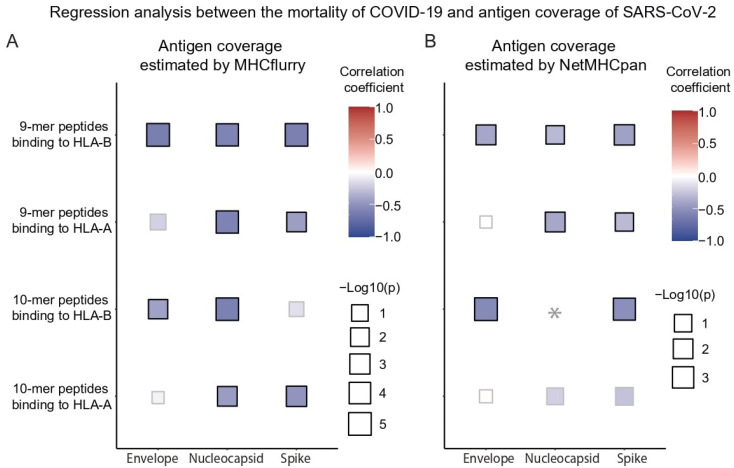
The *p* value and correlation coefficient of linear regression analysis between the mortality of COVID-19 and the antigen coverage of SARS-CoV-2 derived from envelope, nucleocapsid, and spike domains. The bold border indicates *p* value ≤ 0.05. The antigen coverage was estimated by MHCflurry (**A**) and NetMHCpan (**B**). The asterisk indicates the case that no peptides were predicted to be presented by any HLAs.

The number of antigens from the envelope, nucleocapsid, and spike RNA domains of SARS-CoV-2 have been reported to be associated with the death rate of COVID-19 [11]. In the previous study, antigen coverage derived from the envelope and nucleocapsid domains had a positive correlation with the COVID-19 death rate, whereas spike-domain antigens were negatively associated with the mortality rate. We also performed regression analysis between the antigen coverage and mortality rate by dividing the antigens into three domains. The antigen coverage generated from the spike domain correlated negatively with the death rate of COVID-19, as reported in a previous study; however, a negative correlation with the mortality rate was consistently observed for the envelope and nucleocapsid domains, which disagreed with a previous study (Figure 3). Specifically, the consistent negative correlation between antigen coverage and the death rate across the three domains showed statistical significance for the antigens presented by HLA-B, and the correlation was most significant for the spike domain.

### 3.4. Antigens Most Frequently Presented by HLAs

**Figure 4:**
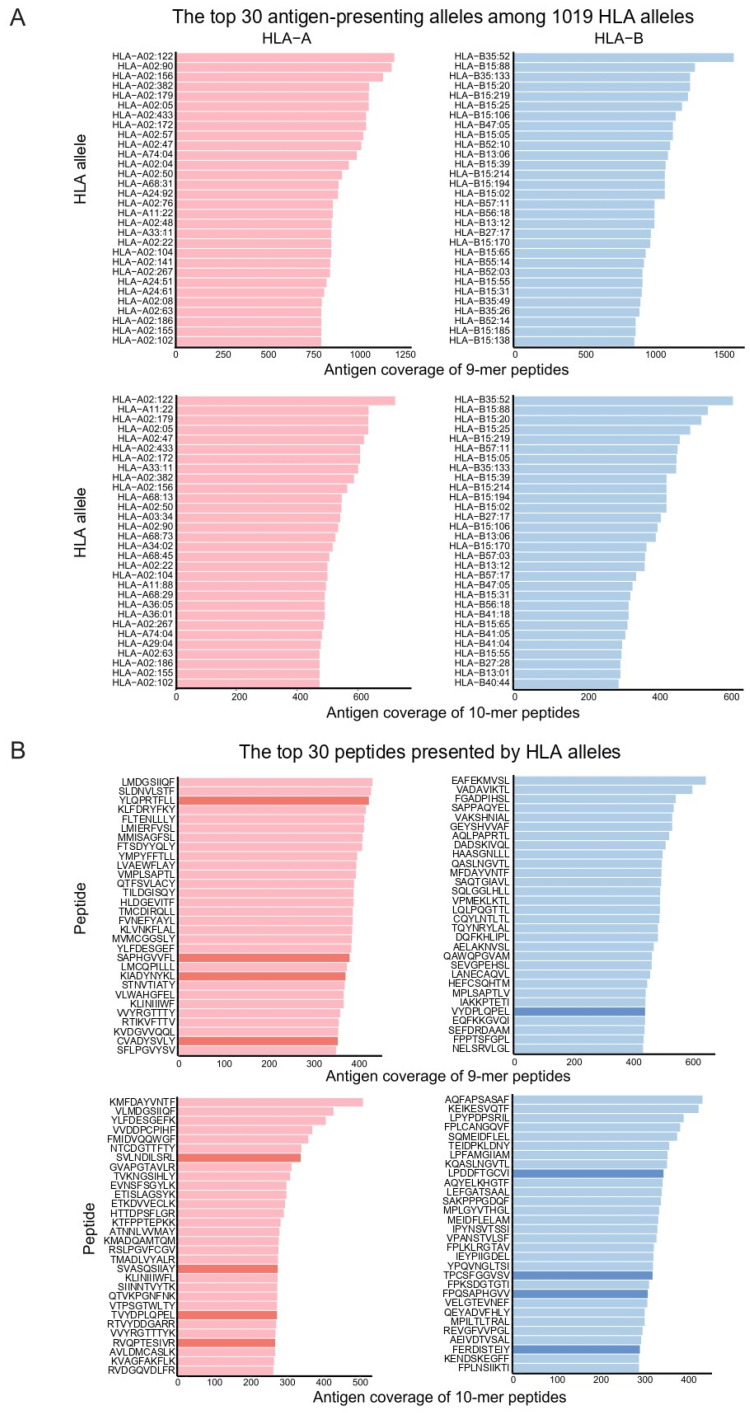
(**A**) The top 30 antigen-presenting alleles in descending order. The red and blue bars indicate the antigen presentation counts for HLA-A and HLA-B, respectively. The antigen coverage in all figures is the result predicted by MHCflurry. (**B**) The top 30 peptides presented by HLA alleles in descending order. The character on the bar indicates the SARS-CoV-2 domain from which the peptide is derived. The red and blue bars indicate the antigen presentation counts for HLA-A and HLA-B, respectively. The bars in a dark color indicate a peptide derived from the spike domain.

Finally, we summarized the most frequently presented SARS-CoV-2 antigens to their corresponding HLAs worldwide. HLAs were ranked by the number of antigens that they are predicted to present, and the top 30 HLA-A and HLA-B alleles overlapped considerably, irrespective of their different predictions for antigen lengths and algorithms (Figure 4A and Figure S2A). We also ranked the top 30 most frequently presented antigens across HLA alleles, lengths, and prediction algorithms (Figure 4B and Figure S2B). In contrast to HLAs, antigens were rarely consistent with each other with regard to their different lengths and predictions. We highlight the antigens derived from the spike domain because they are known for their potential as vaccines for coronavirus family members [14], and they showed strong potential for COVID-19 prognosis in our analysis.

## 4. Discussion

In this study, we provided an outlook on the correlation between the antigen coverage presented by HLAs and the mortality rate of COVID-19. We also compared the effect of antigen coverage on mortality with other risk factors and validated its favorable effect irrespective of other factors. Two types of in silico methods were used to infer antigen presentation to calculate antigen coverage, and the two predictions showed a similar trend regarding the negative correlation between antigen coverage and the death rate of COVID-19, although the MHCflurry prediction exhibited a more robust correlation. This negative correlation was predominantly significant in HLA-B but not in HLA-A. Furthermore, we analyzed their correlation in accordance with the domains of SARS-CoV-2 and discovered a significant negative correlation in spike domain antigen coverage with mortality, which was also mentioned in a previous study [11]. Specifically, it is often reported that coronavirus spike domain antigens may be used for vaccine development [15].

However, this study has a limitation with respect to correlation accuracy due to the inference of the antigen coverage because it was difficult to obtain HLA allele information for individual patients with COVID-19. Therefore, we chose countries that had sufficient COVID-19 cases (*n* > 1000, 12 January 2021) to minimize bias. Additionally, HLA-C was not analyzed among MHC class I alleles because its frequency has been calculated from an insufficient population size in most countries and it has been reported that the cytotoxic T lymphocyte immune response is evoked mainly by HLA-A and HLA-B [16]. Recent studies of sequencing HLA alleles [17] and genomes [18] of individual COVID-19 patients enhance our understanding of the effect of HLA allele and genetic background on the severity of COVID-19 infection although they were focused on a specific population. Other risk factors and environmental elements, such as COVID-19 policies [19], including social distancing, number of PCR tests, and population density, might have been considered in our study, but we chose only eight risk factors with a comparable size for our analysis. We collected the epidemiological data of COVID-19 patients for a relatively early period, and therefore the further study needs to consider the mutation [20] during evolution after the first outbreak in addition to the environmental factors. 

In the previous study, SARS-CoV-2 epitopes of YLQPRTFLL and FLLNKEMYL that were presented by HLA-A*02:01 generated a strong CD8+IFN-γ+ response measured by intracellular cytokine staining assays by in vitro restimulation [21]. Additionally, the SARS-CoV-2-derived HLA-binding peptides of SAPPAQYEL, FTSDYYQLY, KLVNKFLAL, MIAQYTSAL, and CVADYSVLY were identified to be recognized by CD8+ T cells by sorting and sequencing pMHC multimers that were bound to CD8+ T cells [22]. Those seven epitopes were within the top 30 ranks of antigen coverage presented by HLA-A and HLA-B in our study (Figure 4B and Figure S2B). In other previous studies, HLA-B*15:03 and HLA-B*15:21 were predicted to present the most antigens in patients with COVID-19 [23,24]. HLA-B*15:03 was also ranked within the top 10% and 1% as presenting the largest number of antigens in our prediction using MHCflurry and NetMHCpan, respectively (Figure S2A). HLA-B*15:21 was highly ranked (within top 10%) to present the most antigens only in the prediction by MHCflurry. Therefore, the landscape of alleles presenting SARS-CoV-2 antigens seems to be considerably consistent with the previous study although it was not perfectly similar due to discrepancy of prediction methodology or analyzed population. Furthermore, another subtype of HLA-B*15, i.e., HLA-B*15:88, was identified as the novel allele presenting the most SARS-CoV-2 antigens. We focused the effect of HLA class I on SARS-CoV-2-induced mortality. However, a recent study reported the association of HLA class II with the severity of COVID-19 infection [25]. Therefore, a study concerning the antigen coverage presented by MHC class II is needed to better understand SARS-CoV-2-induced mortality if the related data is available. 

## Data Availability

Epidemiological and antigen coverage information is summarized in Table S1.

## Supplementary Materials

The following supporting information can be downloaded at: https://www.mdpi.com/article/10.3390/vaccines10111917/s1, Figure S1: The global correlation between antigen coverage of SARS-Cov-2 predicted by NetMHCpan and mortality of COVID-19; Figure S2: Top antigen-presenting HLA alleles and peptides predicted by NetMHCpan; Supplementary Table S1: Epidemiological and antigen coverage information.
