# Supplementary material for: Antigen Coverage Presented by MHC Class I Has a Negative Correlation with SARS-CoV-2-Induced Mortality
Source: Vaccines (Basel). 2022 Nov 13;10(11):1917. doi: 10.3390/vaccines10111917 (PMC9698063; doi:10.3390/vaccines10111917)
Supplement: Supplementary file 1 [file vaccines-10-01917-s001.zip › vaccines-1954857-supplementary.pdf]

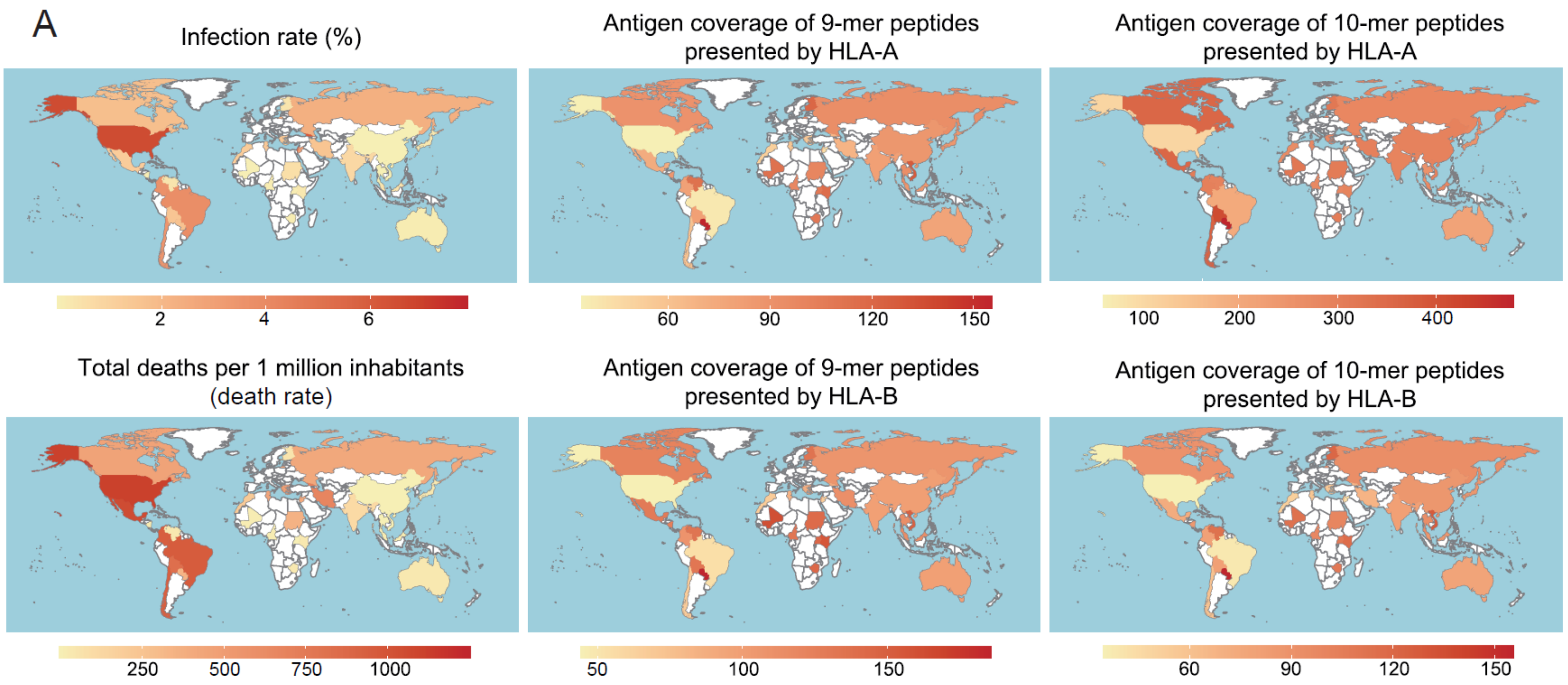
A
Infection rate (%)
2
4
6
Antigen coverage of 9-mer peptides presented by HLA-A
60
90
120
150
Antigen coverage of 10-mer peptides presented by HLA-A
100
200
300
400
Total deaths per 1 million inhabitants (death rate)
250
500
750
1000
Antigen coverage of 9-mer peptides presented by HLA-B
50
100
150
Antigen coverage of 10-mer peptides presented by HLA-B
60
90
120
150

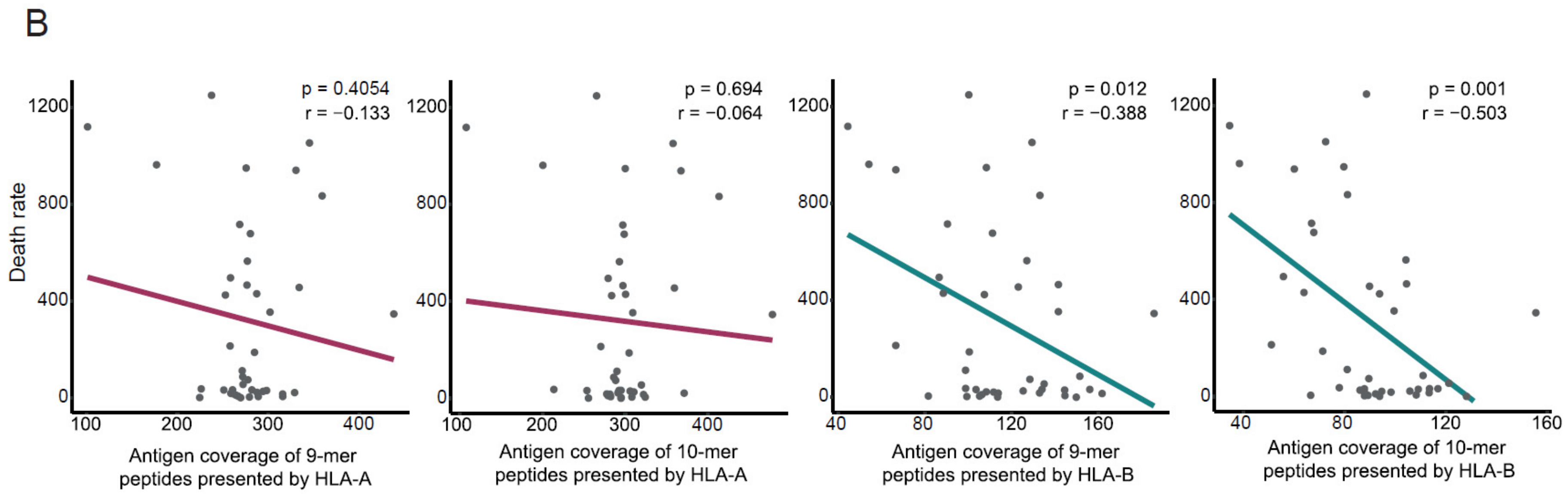

Figure S1. (A) Global heatmaps showed antigen coverage of SARS-CoV-2, infection rate, and mortality of COVID-19 for each country. The antigen coverage in all figures is the result predicted by NetMHCpan.

(B) The results of linear regression of antigen coverage and mortality. The X-axis represents antigen coverage, and the Y-axis represents mortality. The red and blue lines indicate correlation coefficient of mortality with the antigen coverage of HLA-A and HLA-B, respectively.

A

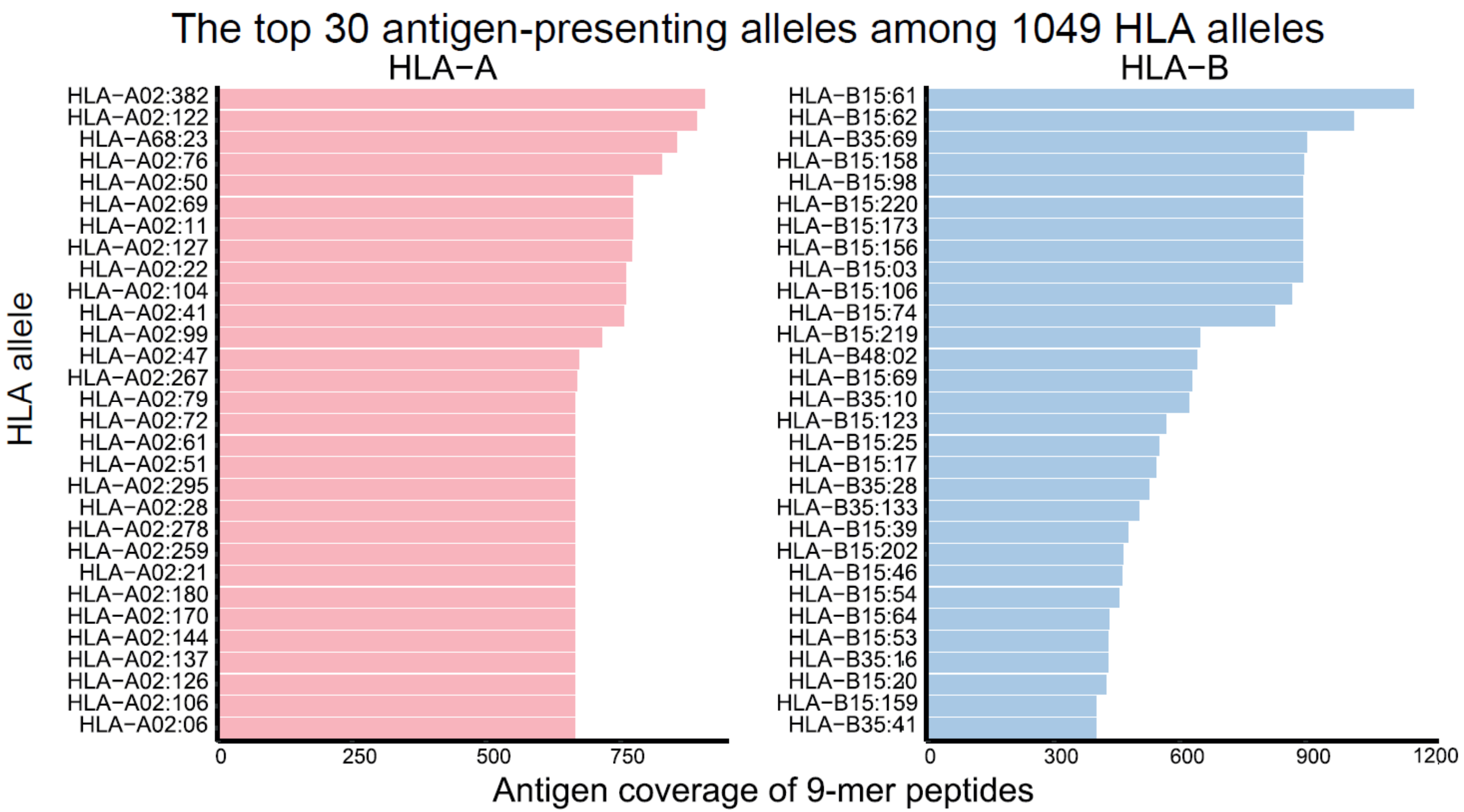

The top 30 antigen-presenting alleles among 1049 HLA alleles
HLA−A
HLA−B
HLA allele
HLA−A02:382
HLA−A02:122
HLA−A68:23
HLA−A02:76
HLA−A02:50
HLA−A02:69
HLA−A02:11
HLA−A02:127
HLA−A02:22
HLA−A02:104
HLA−A02:41
HLA−A02:99
HLA−A02:47
HLA−A02:267
HLA−A02:79
HLA−A02:72
HLA−A02:61
HLA−A02:51
HLA−A02:295
HLA−A02:28
HLA−A02:278
HLA−A02:259
HLA−A02:21
HLA−A02:180
HLA−A02:170
HLA−A02:144
HLA−A02:137
HLA−A02:126
HLA−A02:106
HLA−A02:06
HLA−B15:61
HLA−B15:62
HLA−B35:69
HLA−B15:158
HLA−B15:98
HLA−B15:220
HLA−B15:173
HLA−B15:156
HLA−B15:03
HLA−B15:106
HLA−B15:74
HLA−B15:219
HLA−B48:02
HLA−B15:69
HLA−B35:10
HLA−B15:123
HLA−B15:25
HLA−B15:17
HLA−B35:28
HLA−B35:133
HLA−B15:39
HLA−B15:202
HLA−B15:46
HLA−B15:54
HLA−B15:64
HLA−B15:53
HLA−B35:16
HLA−B15:20
HLA−B15:159
HLA−B35:41
0
250
500
750
0
300
600
900
1200
Antigen coverage of 9-mer peptides

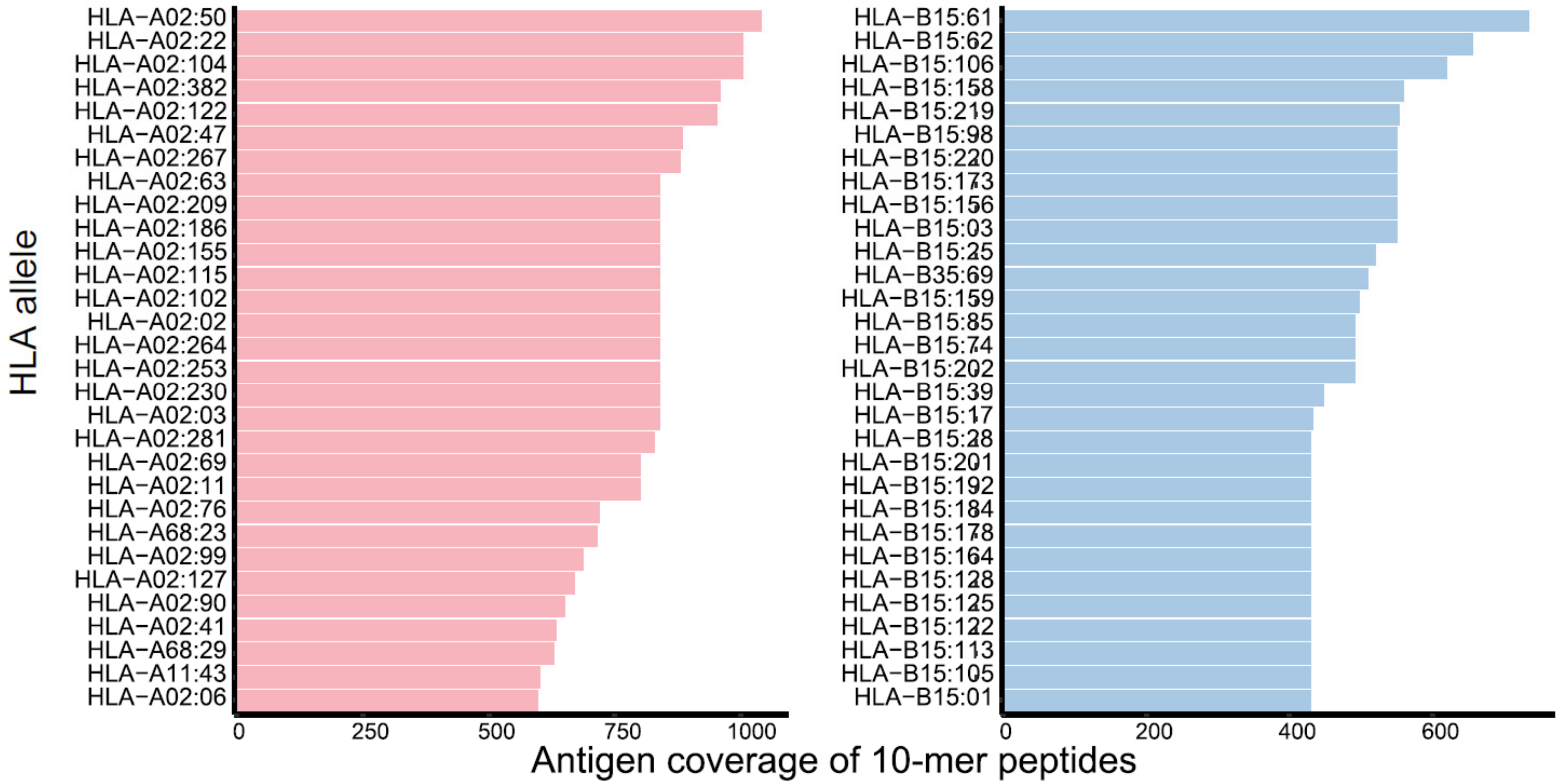

HLA allele
HLA−A02:50
HLA−A02:22
HLA−A02:104
HLA−A02:382
HLA−A02:122
HLA−A02:47
HLA−A02:267
HLA−A02:63
HLA−A02:209
HLA−A02:186
HLA−A02:155
HLA−A02:115
HLA−A02:102
HLA−A02:02
HLA−A02:264
HLA−A02:253
HLA−A02:230
HLA−A02:03
HLA−A02:281
HLA−A02:69
HLA−A02:11
HLA−A02:76
HLA−A68:23
HLA−A02:99
HLA−A02:127
HLA−A02:90
HLA−A02:41
HLA−A68:29
HLA−A11:43
HLA−A02:06
0
250
500
750
1000
HLA−B15:61
HLA−B15:62
HLA−B15:106
HLA−B15:158
HLA−B15:219
HLA−B15:98
HLA−B15:220
HLA−B15:173
HLA−B15:156
HLA−B15:03
HLA−B15:25
HLA−B35:69
HLA−B15:159
HLA−B15:85
HLA−B15:74
HLA−B15:202
HLA−B15:39
HLA−B15:17
HLA−B15:28
HLA−B15:201
HLA−B15:192
HLA−B15:184
HLA−B15:178
HLA−B15:164
HLA−B15:128
HLA−B15:125
HLA−B15:122
HLA−B15:113
HLA−B15:105
HLA−B15:01
0
200
400
600
Antigen coverage of 10-mer peptides

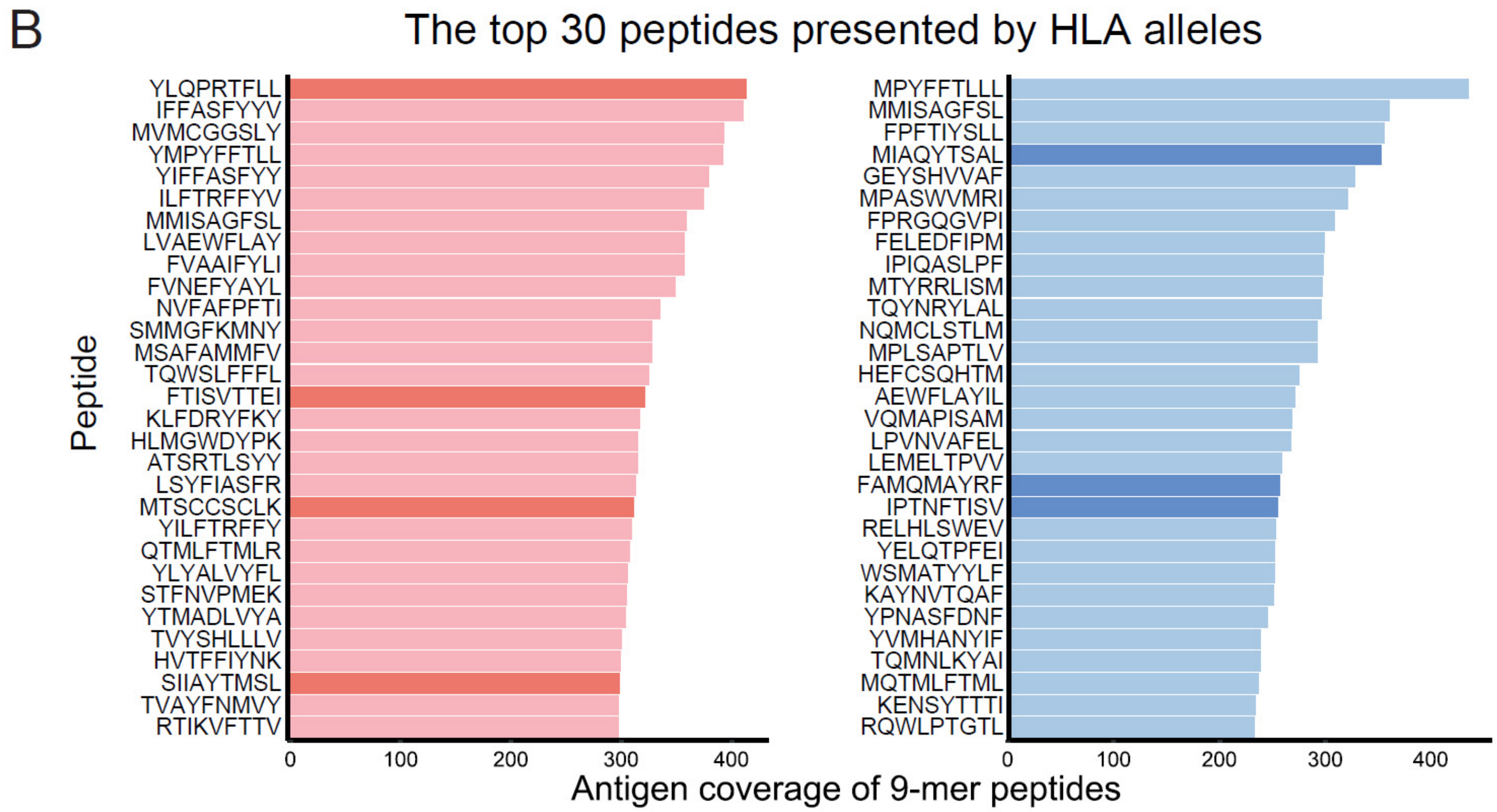

B
The top 30 peptides presented by HLA alleles
Peptide
YLQPRTFLL
IFFASFYYV
MVMCGGSLY
YMPYFFTLL
YIFFASFYY
ILFTRFFYV
MMISAGFSL
LVAEWFLAY
FVAAIFYLI
FVNEFYAYL
NVFAFPFTI
SMMGFKMNY
MSAFAMMFV
TQWSLFFFL
FTISVTTEI
KLFDRYFKY
HLMGWDYPK
ATSRTLSYY
LSYFIASFR
MTSCCSCLK
YILFTRFFY
QTMLFTMLR
YLYALVYFL
STFNVPMEK
YTMADLVYA
TVYSHLLLV
HVTFFIYNK
SIIAYTMSL
TVAYFNMVY
RTIKVFTTV
0
100
200
300
400
MPYFFTLLL
MMISAGFSL
FPFTIYSLL
MIAQYTSAL
GEYSHVVAF
MPASWVMRI
FPRGQGVPI
FELEDFIPM
IPIQASLPF
MTYRRLISM
TQYNRYLAL
NQMCLSTLM
MPLSAPTLV
HEFCSQHTM
AEWFLAYIL
VQMAPISAM
LPVNVAFEL
LEMELTPVV
FAMQMAYRF
IPTNFTISV
RELHLSWEV
YELQTPFEI
WSMATYYLF
KAYNVTQAF
YPNASFDNF
YVMHANYIF
TQMNLKYAI
MQTMLFTML
KENSYTTTI
RQWLPTGTL
0
100
200
300
400
Antigen coverage of 9-mer peptides

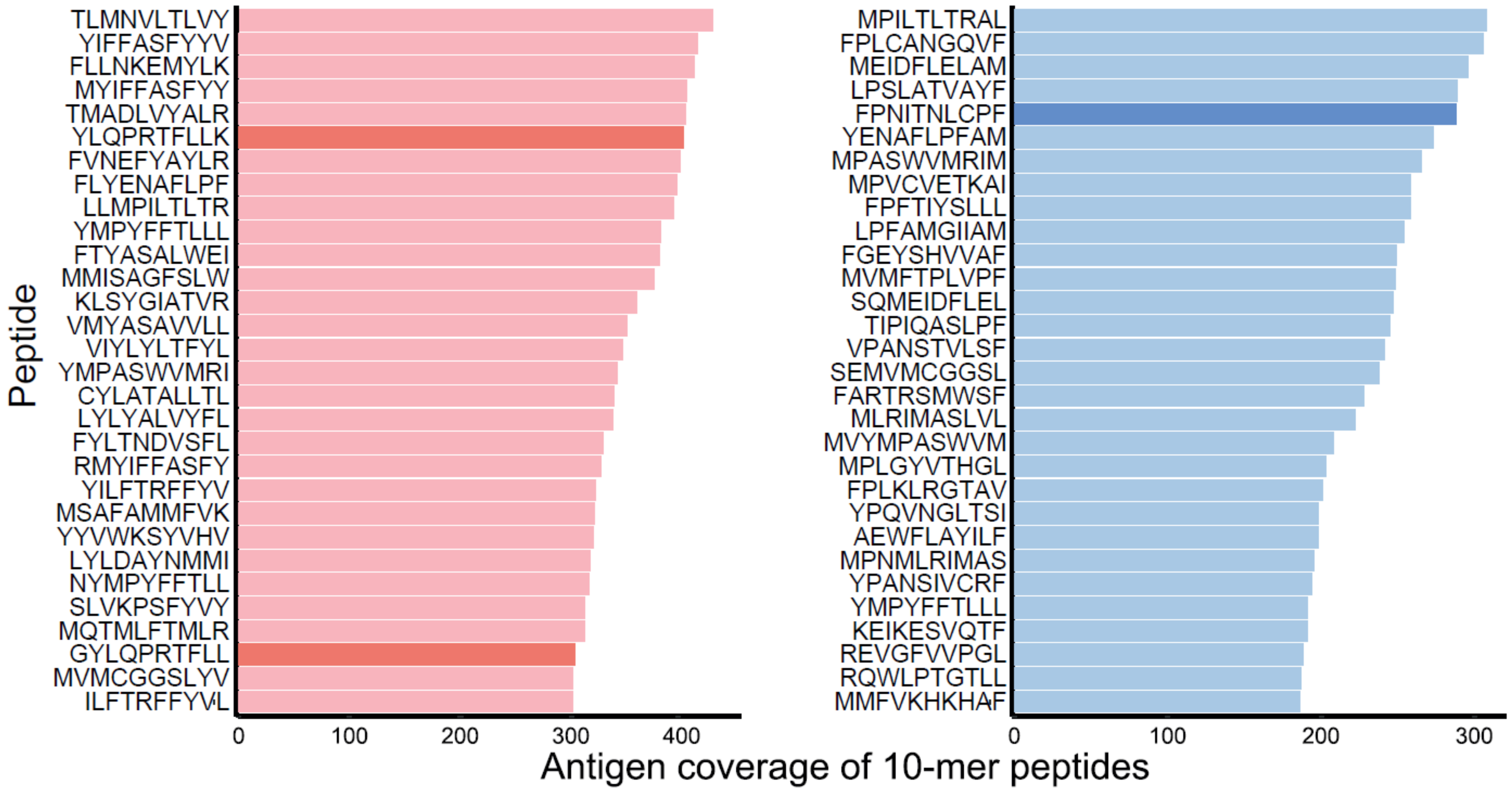

Figure S2. (A) The top 30 antigen-presenting alleles in descending order. The red and blue bars indicate the antigen presentation counts for HLA-A and HLA-B, respectively. The antigen coverage in all figures is the result predicted by NetMHCpan.

(B) The top 30 peptides presented by HLA alleles in descending order. The character on the bar indicates the SARS-CoV-2 domain from which the peptide is derived. The red and blue bars indicate the antigen presentation counts for HLA-A and HLA-B, respectively. The bar in dark color indicates a peptide derived from spike domain.

Table S1. Epidemiological and antigen coverage information (MF: antigen coverage prediction by MHCflurry, nMp: antigen coverage prediction by NetMHCpan)

**Country**	**MF9_A**	**MF9_B**	**MF10_A**	**MF10_B**	**nMp9_A**	**nMp9_B**	**nMp10_A**	**nMp10_B**	**infection rate**	**death rate**	**Asthma rate**	**Chronic kidney disease rate**	**Hypertension rate**	**Liver disease rate**	**Obesity rate**	**Smoking rate**	**Type 1 diabetes rate**	**Type 2 diabetes rate**
Australia	505.966638	500.120595	269.089204	188.753671	226.144674	98.6610674	213.655449	77.8905618	0.1154315957	36.6942894	8.91	10.83	33.7846874	16.89	29	14.9	0.77	5.69
Bolivia	526.107402	316.740313	244.545173	98.6062944	358.909756	132.898829	413.093024	81.1406342	1.5406831547	833.743919	4.89	6.16	27.2979047	14.12	20.2	23.8	0.18	3.73
Brazil	480.446735	265.3317	258.872063	85.3099424	177.136269	54.0565385	200.439692	38.5015	3.8347022597	962.146788	4.57	8.35	33.4706273	24.31	22.1	15.3	0.38	5.7
Cameroon	465.343658	362.73542	259.319241	133.064317	258.664185	132.729967	277.50125	98.2958696	0.1087922256	18.1536491	2.42	4.7	18.1970942	19.77	11.4	22.35	0.18	1.85
Canada	497.322342	329.286053	235.415399	114.413022	333.705932	122.935831	359.211119	89.8222373	1.7656356551	455.512259	4.81	10.87	23.3108917	13.06	29.4	14.95	1.14	5.91
Chile	472.175	214.7	276.925	61.3	330.15	66.475	367.1	60.15	3.5275904124	939.484731	5.78	10.15	29.9088357	13.72	28	38	0.4	7.96
China	471.262275	392.018984	246.086537	134.627582	279.072884	99.2159141	294.896031	87.8349001	0.0062581987	3.31089867	1.82	10.15	34.0398121	31.2	6.2	24.7	0.14	6.61

Colombia	439.921386	292.111759	226.688451	100.99548	275.473302	108.215677	299.84798	79.6834204	3.6705724799	948.724177	3.41	11.47	28.7	19.84	22.3	11.1	0.17	7.81
CostaRica	449.159539	331.149892	237.374791	116.902273	276.394539	141.416224	297.02745	104.422412	3.5858442191	465.355824	5.51	14.75		18.37	25.7	13.4	0.16	8.43
Czech	403.114091	322.52514	210.236846	113.780343	237.45495	100.11047	265.2494	88.61224	7.8629114609	1249.10002	3.22	12.81	45.3197537	18.17	26	33.2	0.51	15.23
Finland	434.643357	324.651047	228.885115	117.494517	272.055	134.834	319.374	121.085	0.3582786287	55.1516435	7.3	10.22	50.478437	12.94	22.2	20.85	1.17	11.04
Georgia	447.00084	332.051175	230.644951	105.970919	268.404743	90.2797286	296.886814	66.9563857	6.1369838892	715.614603	1.99	13.17	40.6359441	23.44	21.7	31.7	0.4	9.88
Greece	425.58993	298.247552	210.633667	95.3355949	258.304271	86.4415103	279.192155	55.8495555	1.3622079480	495.329522	5.99	14.67	37.7660521	17.11	24.9	42.65	0.76	8.67
HongKong	479.117241	409.002995	269.881839	146.700085	328.67347	111.5631	370.98479	94.63143	0.1249581532	21.4029369			29.1859275					
India	413.192034	394.380168	225.796942	142.5896	271.152582	98.62075	289.840176	81.0164873	0.7731671013	111.661853	2.53	8.49	27.4590874	18.1	3.9	11.15	0.26	6.03
Iran	443.662776	331.715043	228.56295	107.980153	280.112195	111.028659	298.563293	67.8451463	1.5521645016	677.753619	3.26	10.37	26.1517972	34.57	25.8	11.1	0.43	6.22
Israel	444.315	311.565	229.095	123.26	287.09	88.375	300.24	63.96	5.6946882934	429.610543	5.4	8.45	37.0522854	19.59	26.1	30.25	0.42	5.97
Japan	423.166308	367.98129	207.496331	115.829423	250.938785	103.425874	253.616268	87.8006322	0.2273103448	32.0570749	4.18	20.18	41.9302368	15.99	4.3	22.15	0.45	7.16
Jordan	410.701613	340.520619	210.327957	102.599656	201.446	65.448	206.337	43.257	3.1019299457	406.308608	4.32	7.84	27.8361088	32.27	35.5	40.45	0.31	5.43

Kenya	496.565979	378.525416	266.391162	130.885328	281.771678	155.878689	292.259618	116.804788	0.1869194858	32.5255997	2.94	3.78	35.7606691	16.69	7.1	13.35	0.21	1.58
Malaysia	437.460935	442.991601	233.94928	152.00423	261.029396	113.661635	281.258557	87.8516308	0.4244118850	17.1959342	2.67	10.54	25.8306251	27.37	15.6	22.2	0.1	6.12
Mali	465.586935	380.050302	239.648241	116.492958	315.561	161.566	321.89	113.339	0.0397745287	15.595802	2.25	3.49	32.3474098	18.97	8.6	20	0.15	1.59
Mexico	502.62312	299.435501	254.341891	88.5136652	344.974903	129.247594	357.540671	72.5081505	1.2040320754	1052.34974	2.85	13.81	28.873739	21.8	28.9	13.7	0.14	10.13
Morocco	413.534129	300.905728	215.59572	116.880454	257.857439	66.4506543	270.362584	51.1157732	1.2503714584	213.94346	2.96	9.64	33.2397983	32.88	26.1	23.4	0.37	6.52
NewZealand	506.322321	257.654018	253.240708	116.655799	315.6249	81.4605126	307.845683	66.5938844	0.0392875355	5.26360336	6.46	11.08	33.5540292	13.86	30.8		0.79	5.09
Nicaragua	451.511443	344.128614	233.60597	107.239282	294.04	125.158	310.27	86.072	0.0774413394	26.4131135	4.18	10.79	25.8129606	19.88	27.6		0.13	6.44
Paraguay	524.455823	399.114152	269.498978	155.703093	437.561217	185.519565	477.526826	155.42587	1.6677908419	346.097121	5.73	7.55	27.5008292	18.22	20.3	18.1	0.27	4.5
Phillippines	502.65	488.49	246.5	190.39	271.71	151.29	285.95	110.94	0.4499026744	86.762793	4.86	9.54	25.7171627	22.94	6.4	25.75	0.1	3.19
Russia	418.230243	331.682442	214.631715	118.886298	252.69689	107.29214	283.412852	93.7826851	2.3333889488	424.221076	2.51	15.55	40.8433118	20.92	23.1	40.9	0.56	4.34
SaudiArabia	457.2536	331.872606	242.752924	112.114099	284.681707	100.34186	304.480809	71.3628909	1.0842381864	187.486907	2.48	8.47	26.1629456	40.76	35.4	15.4	0.63	7.23
Singapore	474.702876	399.731672	263.597871	138.73987	288.795773	105.092198	323.917142	89.2235142	1.0170581300	5.00699166	3.71	13.86	23.857377	16.25	6.1	16.5	0.45	8.15

SouthAfrica	440.695303	372.226872	236.912912	131.214347	276.812734	126.862453	292.777943	104.171545	2.0954428567	564.236284	4.13	7.28	46.4220369	24.96	28.3	18.95	0.28	4.95
SouthKorea	463.785586	338.949932	234.600909	118.31902	287.35894	108.15956	291.03649	105.73693	0.1354099666	22.4680412	3.75	10.83	25.9986651	15.77	4.7	27	0.47	7.79
SriLanka	414.348651	401.802198	233.261738	149.89011	264.157	106.124	279.021	92.211	0.2309303356	11.073964	3.82	13.49	24.4483799	22.61	5.2	14.4	0.1	11.2
Sudan	488.301288	353.633302	242.912785	123.228251	301.701	141.412	308.919	99.499	0.5616754096	353.63677	4.2	5.61	27.9095306	20.08	6.6		0.3	4
Swiss	458.019231	350.595934	194.867692	141.308549	79.5634131	84.599773	63.0948119	71.898045	5.5665040659	883.072329	7.34	11.44	35.0564256	13.82	19.5	23.3	0.88	7.74
Thailand	488.884472	422.554167	273.839752	143.290625	224.496	113.398	255.445	93.793	0.0158782301	1.00866731	3.39	16.67	23.606569	31.52	10	21.85	0.11	6.98
Tunisia	423.527363	339.568123	227.348259	114.587404	262.48	84.427	280.829	57.853	1.3661002057	447.287261	3.74	11.77	23.0968044	35.72	26.9		0.43	9.25
UAE	473.928356	373.230734	249.455928	116.731354	277.359	128.26042	288.29832	89.5487	2.4165510765	74.2012752	6.84	12.94	18.2292917	44.07	31.7		0.64	8
Uganda	466.477171	376.403804	252.686048	130.844833	266.547271	144.436542	282.238208	108.243542	0.0852530382	6.79910034	5.33	2.98	28.6604128	13.36	5.3	9.65	0.16	1.77
USA	448.122662	352.284511	239.348466	122.354369	101.407302	44.3531065	107.823301	34.623459	6.6675982223	1118.78774	10.92	12.94	31.4609323	15.14	36.2	17.25	0.86	11.64
Venezuela	471.918	365.198198	216.121	120.783784	260.557	133.86	294.792	113.479	0.3587634944	32.7659047	3.86	12.28	28.2992847	22.14	25.6		0.15	8.02
Vietnam	469.105088	463.87567	263.960053	171.192666	269.41117	149.650661	294.691158	128.077843	0.0015736576	0.36379139	3.06	10.74	20.9534693	25.72	2.1	24.2	0.09	4.68

Zimbabwe	480.178715	356.10161	254.235944	125.930584	297.893	144.307	305.726	109.044	0.1269831998	29.9764782	1.88	4.88	32.5792285	21.84	15.5	16.65	0.21	3.3
